# A novel mutual information-based Boolean network inference method from time-series gene expression data

**DOI:** 10.1371/journal.pone.0171097

**Published:** 2017-02-08

**Authors:** Shohag Barman, Yung-Keun Kwon

**Affiliations:** School of Electrical Engineering, University of Ulsan, Daehak-ro, Nam-gu, Ulsan, Republic of Korea; Instituto Nacional de Medicina Genomica, MEXICO

## Abstract

**Background:**

Inferring a gene regulatory network from time-series gene expression data in systems biology is a challenging problem. Many methods have been suggested, most of which have a scalability limitation due to the combinatorial cost of searching a regulatory set of genes. In addition, they have focused on the accurate inference of a network structure only. Therefore, there is a pressing need to develop a network inference method to search regulatory genes efficiently and to predict the network dynamics accurately.

**Results:**

In this study, we employed a Boolean network model with a restricted update rule scheme to capture coarse-grained dynamics, and propose a novel mutual information-based Boolean network inference (MIBNI) method. Given time-series gene expression data as an input, the method first identifies a set of initial regulatory genes using mutual information-based feature selection, and then improves the dynamics prediction accuracy by iteratively swapping a pair of genes between sets of the selected regulatory genes and the other genes. Through extensive simulations with artificial datasets, MIBNI showed consistently better performance than six well-known existing methods, REVEAL, Best-Fit, RelNet, CST, CLR, and BIBN in terms of both structural and dynamics prediction accuracy. We further tested the proposed method with two real gene expression datasets for an *Escherichia coli* gene regulatory network and a fission yeast cell cycle network, and also observed better results using MIBNI compared to the six other methods.

**Conclusions:**

Taken together, MIBNI is a promising tool for predicting both the structure and the dynamics of a gene regulatory network.

## Background

A gene regulatory network (GRN) consists of various molecular components such as genes, proteins, and mRNA, and their genetic interactions. Discovering the genetic interactions from a time-series gene expression dataset is considered to be a network inference or reverse engineering problem. Specifically, the problem aims to infer not only a set of regulatory genes for a target gene (i.e., a network structure inference), but also the regulatory rules between genes (i.e., a network dynamics inference). Many methods based on various computational models including a relevance network [1], a Bayesian network [2], and a differential equation [3] have been proposed for the reverse engineering problems. Another popular computational model is a Boolean network (BN), wherein the state of a gene is represented by a Boolean value. This value is updated by a logical rule based on multiple regulatory genes, and some BN-based inference methods have been proposed [4, 5]. Although a BN model is basically attractive for inferring a relatively large GRN due to the simple representation, the previous BN-based methods were not suitable for large-scale network inference, because all possible combinations of genes were exhaustively compared so as to find a best set of regulatory genes for a given target gene. For example, a best-fit algorithm [6] exhaustively searches regulatory genes with all possible combinations for a target gene and finds the best fitting function that minimizes the errors. On the other hand, some other BN-based methods have limited the maximum number of regulatory genes to reduce a computation at cost. For example, a Bayesian inference approach for a Boolean network (BIBN) [7] where the maximum number of regulatory genes was bounded by two was used to infer a Boolean network by maximizing a joint posterior probability. Therefore, a way to select the most informative regulatory genes efficiently is required, and herein, we incorporate an approximated multivariate mutual information measure to resolve this problem.

Mutual information, which is a measure used to represent a mutual dependency between two random variables, can describe the dependency of a given target gene on a set of candidate regulatory genes. In this regard, many approaches based on mutual information have been proposed for reverse engineering problems. The first approach was the reverse engineering algorithm REVEAL, which searches for a set of genes that maximizes a discrete mutual information value for a target gene [8]. A method called a path consistency algorithm based on conditional mutual information (PCA-CMI) employs conditional mutual information to detect nonlinear statistical dependencies between gene pairs and distinguish direct interactions from indirect interactions [9]. The time-delay algorithm for the reconstruction of accurate cellular networks (ARACNE) method is used to compute the mutual information by considering a time gap between gene expression values [10]. The final example is the mutual information distance and entropy reduction (MIDER) method, which defines a mutual information-based distance between genes to specify the directionality [11]. These mutual information-based methods are computationally expensive, because they are implemented to compute exact mutual information values over all possible combinations of genes. To reduce the computational cost, other mutual information-based methods have been proposed. For example, the relevance network (RelNet) algorithm [12] computes pairwise mutual information for a target gene and infers an interaction if it is larger than a pre-specified threshold. The context likelihood of relatedness (CLR) method [13] reduces the connections from a complete graph by discarding false connections through comparisons of pairwise mutual information scores with a background correction of mutual information scores. A chi-square test (CST) [14] was also used to identify a connection by testing whether there was a significant difference between the distributions of a target gene and candidate regulator genes. We note that RelNet, CLR, and CST are computationally effective because they assess only pairwise interactions. Another common limitation is that they are intended to infer only a regulatory structure without any consideration of inferring a regulatory rule or function. Therefore, there is a need to develop a novel approach that cost-efficiently computes the multivariate mutual information and properly extends it to infer regulatory rules for the prediction of network dynamics.

To resolve these limitations, we propose a mutual information-based Boolean network inference (MIBNI) algorithm. The algorithm first employs a mutual information-based feature selection (MIFS) method [15] that was originally suggested for efficient supervised learning in artificial neural networks. We used MIFS to approximate the mutual information between a target gene and a set of candidate regulatory genes to reduce the computational cost. The loss of information increases as the number of regulatory genes considered in MIFS is increased, which can eventually cause a significant decrease in the inference accuracy. To overcome this problem with MIFS, we further devised a SWAP subroutine, which is a greedy algorithm wherein a gene in the set of regulatory genes selected by MIFS is iteratively swapped with another gene in the set of unselected genes. MIBNI was implemented to repeat the MIFS and SWAP subroutines by turns until the desired number of most informative regulatory genes with the highest dynamics accuracy is found. To validate the performance of MIBNI, we compared it with six well-known reverse engineering methods, REVEAL, Best-Fit, RelNet, CST, CLR and BIBN. Through extensive simulations on artificial gene expression datasets, it was shown that MIBNI performed significantly better than the six previous methods in terms of the accuracies of both structural and dynamics inference, even with a relatively small running time. Our method was also tested with two real gene expression datasets related to an *Escherichia coli* gene regulatory network and a fission yeast cell cycle network, and it showed significantly better performance than did any of the previous methods. These successful results indicate that MIBNI is a useful mutual information-based method that can infer a Boolean network from various gene expression datasets.

## Methods

A Boolean network is the simplest model to represent a biological network. In this section, we explain the basic concepts employed in our proposed method to infer a Boolean network from time-series gene expression data.

### A Boolean network model

A Boolean network [16] ***G***(***V***,***A***) consists of a set of nodes ***V*** = {***v***_**1**_,***v***_**2**_,…,***v***_***N***_} and a set of interactions ***A*** = {(***v***_***i***_,***v***_***j***_)|***v***_***i***_,***v***_***j***_ **ϵ *V***}. Every node is represented by a Boolean variable whose state is updated by a Boolean function. Specifically, the value of a Boolean variable ***v***_***i***_ at time ***t*** + **1** is determined by the values of other variables $v_{i_{1}},v_{i_{2}},\ldots ..,v_{i_{k}}$ having a link to ***v***_***i***_ at time ***t*** by the following Boolean function:

$$v_{i}(t+1)=f_{i}(v_{i_{1}}(t),\ldots ,v_{i_{k}}(t)).$$

In this work, it is assumed that each update function is represented by a conjunction (AND rule) or a disjunction (OR rule) and that all of the variables are synchronously updated.

### Boolean network inference problem

**Fig 1:**
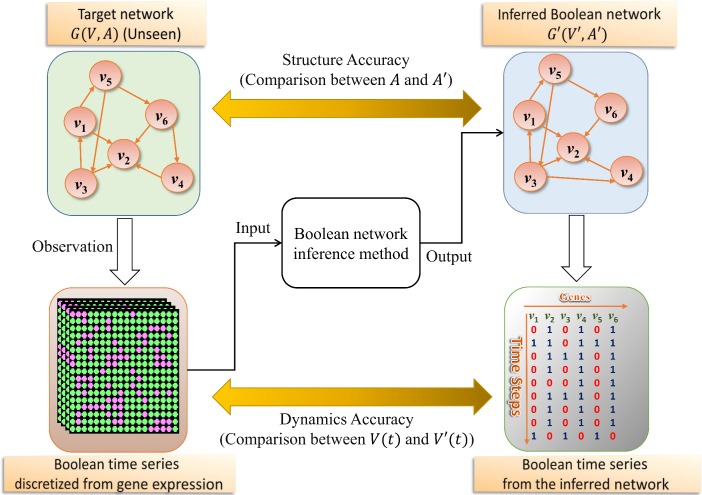
Overview of a Boolean network inference problem. An unseen target network *G*(*V*,*A*) produces a time-series gene expression dataset that is converted to a Boolean time-series dataset by a discretization method. An inference algorithm trains the Boolean dataset as an input and infers a Boolean network *G*′(*V*′,*A*′) as an output. The inference performance is evaluated by a structural accuracy comparing the inferred connections *A*′ to the true connections *A* and by a dynamics accuracy comparing the predicted Boolean time-series data *V*′(*t*) to the observed data *V*(*t*).

Boolean network inference is the problem of inferring a Boolean network that best fits observed time-series gene expression data. The overall approach to that problem is in Fig 1. As shown in the figure, a time-series dataset of real values is obtained from an unseen target network *G*(*V*,*A*). Then, it is transformed into a dataset of binary values by a discretization method. We used the *K*-means clustering algorithm-based discretization method [17]. This method divides all of the expression values of a gene into two clusters, and assigns 1 (the ‘on’ state) and 0 (the ‘off’ state) to the clusters having relatively higher and lower average values, respectively. A Boolean network inference method uses the Boolean dataset as an input and outputs an inferred network *G*′(*V*′,*A*′) as a result. Then, two performance types are evaluated. One is the structural performance evaluated by comparing the structures *G*(*V*,*A*) and *G*′(*V*′,*A*′). For a reliable comparison of the structural performance, we use three well-known metrics, precision, recall, and structural accuracy, as in previous studies [9, 18]. Precision is defined as the percentage of correctly inferred connections out of all predictions, as follows:

$$Precision=\frac{TP}{TP+FP},$$

where *TP* (true positive) and *FP* (false positive) denote the numbers of correctly and incorrectly predicted connections, respectively. Recall is the percentage of inferred connections among the true connections in *G*(*V*,*A*):

$$Recall=\frac{TP}{TP+FN},$$

where *FN* (false negative) means the number of non-inferred connections in *G*(*V*,*A*). Structural accuracy is the percentage of correct predictions, as follows:

$$Structural\;Accuracy=\frac{TP+TN}{TP+FP+FN+TN},$$

where TN (true negative) is the number of correct negative predictions.

The other performance measure is the network dynamics accuracy, wherein we compare the two trajectories of the target and the inferred networks. To specify this, we first define the gene-wise dynamics consistency between the observed variable *v*(*t*) and the predicted variable *v*′(*t*), *E*(*v*,*v*′), as follows:

$$E(v,v^{\prime })=\frac{\sum_{t=2}^{T}I(v(t)=v^{\prime }(t)))}{T-1},$$

where *T* is the number of time steps and *I*(∙) is an indicator function that returns 1 if the condition is true, or 0 otherwise. Then, the network dynamics accuracy is obtained by simply averaging the gene-wise dynamics consistency over all genes as follows:

$$Dynamics\;Accuracy=\frac{\sum_{i=1}^{N}E(v_{i},{v_{i}}^{\prime })}{N},$$

where *N* is the number of genes. In other words, the network dynamics accuracy represents the ratio of the equivalent Boolean values between all pairs of the target and the inferred variables over all time steps except the initial time. Previous studies of the network inference problem have mainly focused on structural measures [19–21], and the dynamics performance has not frequently been taken into account. The dynamics performance measure is more crucial, considering that the network inference ultimately aims to characterize various cellular dynamical behaviors through complex molecular interactions.

### Mutual information

Our approach includes a selection of regulatory variables that is based on some concepts of information theory. First, the entropy *H*(*X*) of a discrete random variable (gene) *X* is defined to measure the uncertainty of *X* as follows:

$$H(X)=-\sum_{x\in X}p(x)\;\log\;p(x).$$

In addition, the joint entropy *H*(*X*,*Y*) of two discrete random variables *X* and *Y* with a joint probability distribution *p*(*x*,*y*) is defined as follows:

$$H(X,Y)=-\sum_{x\epsilon X}\sum_{y\epsilon Y}p(x,y)\;log\;p(x,y).$$

Finally, we used the mutual information of two discrete variables as follows:

I(X;Y)=H(X)+H(Y)−H(X,Y).

The larger the mutual information is, the more the variables (genes) are dependent on each other.

In this work, we propose a mutual information-based Boolean network inference method called MIBNI. We first introduce two main subroutines, MIFS and SWAP, which are a mutual information-based feature selection procedure and an iterative variable replacement procedure, respectively.

### MIFS and SWAP subroutines

**Fig 2:**
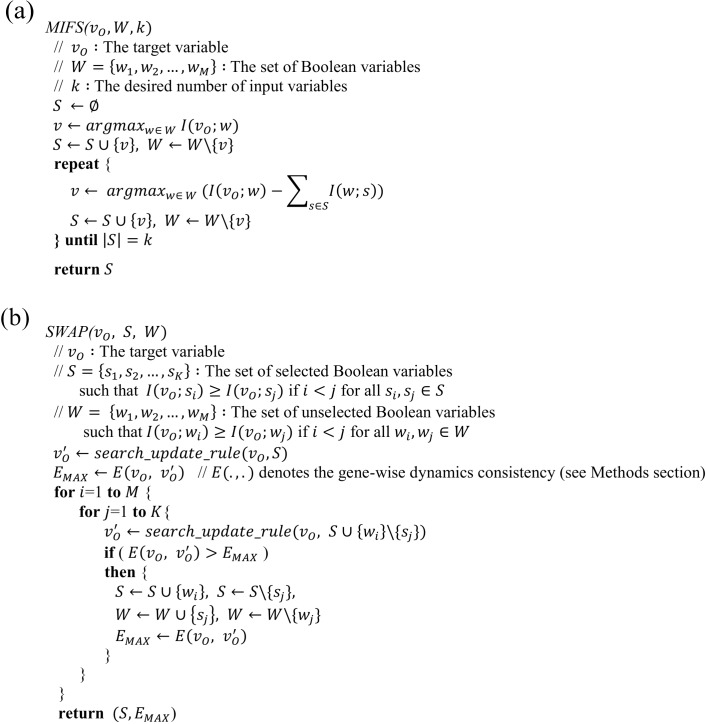
Pseudocodes of two main subroutines in MIBNI. **(a)** MIFS subroutine. This returns *k* most informative variables among *W* for a given target variable *v*_0_. **(b)** SWAP subroutine. This improves the dynamics accuracy by iteratively swapping a variable from the set of selected variables *S* with another variable from the set of unselected variables *W*.

In a previous study [15], MIFS which is a feature selection method based on mutual information was suggested to retrieve the most informative input variables in a generalized supervised learning problem. In this work, we used the method to search for a set of the most informative *k* variables, *S*, among the total set of candidate Boolean variables *W* = {*w*_1_,*w*_2_,…,*w*_*M*_} for a target variable *v*_*O*_ (Fig 2A). The set *S* is initialized with a variable *v* ∈ *W* that maximizes the mutual information with *v*_*O*_. Then, the subroutine selects a next variable *w* among the remaining candidate variables that maximizes *I*(*v*_*O*_;*w*) − ∑_*s*∈*S*_*I*(*w*;*s*) where the second term represents the approximate dependency of *w* to the set of already selected variables *S*. This selection for the next variable is repeated until the desired number of variables (*k*) has been chosen.

To reduce the computational cost, the MIFS subroutine does not compute the exact multivariate mutual information, and it often fails to find the optimal set of regulatory variables. To overcome this problem, we propose a simple iterative SWAP subroutine to improve the dynamics accuracy by swapping the same number of variables between the sets of selected and unselected variables. The pseudo-code of the proposed SWAP subroutine is shown in Fig 2B. Given two sets of selected and unselected variables *S* and *W*, each is assumed to be ordered by the mutual information with the target variable. Every pair of variables *s* ∈ *S* and *w* ∈ *W* is examined to see if swapping *s* and *w* increases the gene-wise dynamics consistency. In this process, it is required to specify the update rule of $v_{O}^{\prime }$ corresponding to the target variable *v*_*O*_ from a set of selected input variables *S* (“*search_update_rule*” in Fig 2B). Since every variable in *S* can be positively or negatively related to *v*_*O*_, there can be 2^|*S*|+1^ conjunction or disjunction update rules. Thus, we chose the update rule that maximizes the gene-wise dynamics consistency among the possible update rules.

### Overall framework of the proposed Boolean network inference algorithm

**Fig 3:**
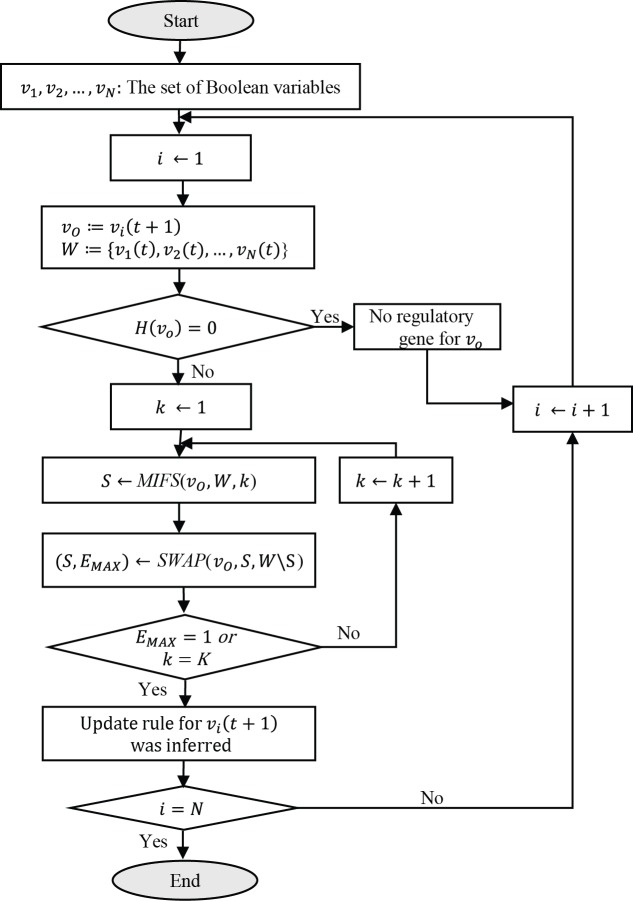
Overall framework of the MIBNI algorithm. Every Boolean variable with a single-step time lag (*v*_*i*_(*t* + 1)) is specified as a target variable. If the entropy value of the target variable is zero, it means that there is no regulatory gene. Otherwise, MIBNI selects an initial set of *k* most relevant variables (*S*) among *W* using the MIFS subroutine for a given a target variable *v*_*O*_ and a set of candidate Boolean variables *W* = {*v*_1_,*v*_2_,…,*v*_*N*_}. Then, some variables in *S* can be replaced with the same number of other variables in *W*\*S* using the SWAP subroutine to improve the identification of input variables. The process is repeated by increasing *k* until an optimal set *S* is found or until *k* equals a parameter *K*.

The overall framework of the proposed MIBNI is shown in Fig 3. This framework considers each variable of one time step lag as a target variable. Given a target variable *v*_*O*_ and a set of candidate Boolean variables *W* = {*v*_1_,*v*_2_,…,*v*_*N*_}, the entropy value of *v*_*O*_ is first examined. When it is zero, it implies that there is no regulatory genes because the value of *v*_*O*_ was fixed all the time steps; otherwise, the subroutine MIFS is called to select the *k* most informative variables with *v*_*O*_. Next, a greedy subroutine SWAP is performed to increase the gene-wise dynamics consistency by swapping the same number of variables between *S* and *W*\*S*. This process is repeated by increasing *k* until an optimal set *S* (i.e., the perfect gene-wise dynamics consistency) is found or *k* equals a parameter *K* which specifies the maximum number of incoming links to be inferred. Considering the computation time, we set *K* to 10 in this study and note that the parameter was set to a relatively small value, two or three in previous studies [6–8] based on Best-Fit, BIBN, and REVEAL. In conclusion, MIBNI searches for a conjunction or disjunction Boolean function that best approximates *v*_*O*_(*t* + 1) using the set of selected input variables *S* for every variable.

## Results and discussion

To validate our approach, we tested it with artificial and real gene expression datasets. We present the results in the following subsections.

### Performance on artificial networks

#### Artificial gene expression data

To test MIBNI, we generated random networks and gene expression datasets by using two different models. In the first model, ten groups of random Boolean networks with different network sizes (|V| = 10,20,…,100 and |A| = 2 ∙ |V|) are created by using the Barabási-Albert (BA) model [22] (see S1 Fig for the pseudo-code). For each group, 30 networks were generated and therefore a total of 300 BA random networks were tested. The state of each gene (node) was randomly initialized between 0 and 1 and it was updated over some time steps by an update function selected uniformly and randomly between the conjunction and the disjunction functions. We note that no discretization method was required for this dataset. In the second random network model, ten groups of random networks with different network sizes (|V| = 10,20,… 100 and variable |A|) are created by using GeneNetWeaver (GNW) [23] software which randomly constructs network structures by extracting modules from known *E. coli* or *S. cerevisiae* gene regulatory networks (herein, the former was selected). The number of links (|A|) is variable because it is determined by GNW in a probabilistic way (see S1 Table for the ranges of the number of links). For each group, 30 networks were generated and therefore a total of 300 GNW random networks were tested. The continuous-valued gene expression data was induced from both of stochastic and ordinary equations and it was converted to Boolean-valued data by using the *K*-means clustering algorithm-based discretization method. In both of BA and GNW random networks, we set the maximum time step (T) to |V| + 10. Then the trajectory of all the Boolean gene values was used as an input to train the MIBNI. After MIBNI learns the input data, it outputs an inferred network structure and predicts time-series Boolean values of all genes. Accordingly, we analyzed the performance of MIBNI in terms of both structural and dynamics accuracies.

#### Structural accuracy analysis

**Fig 4:**
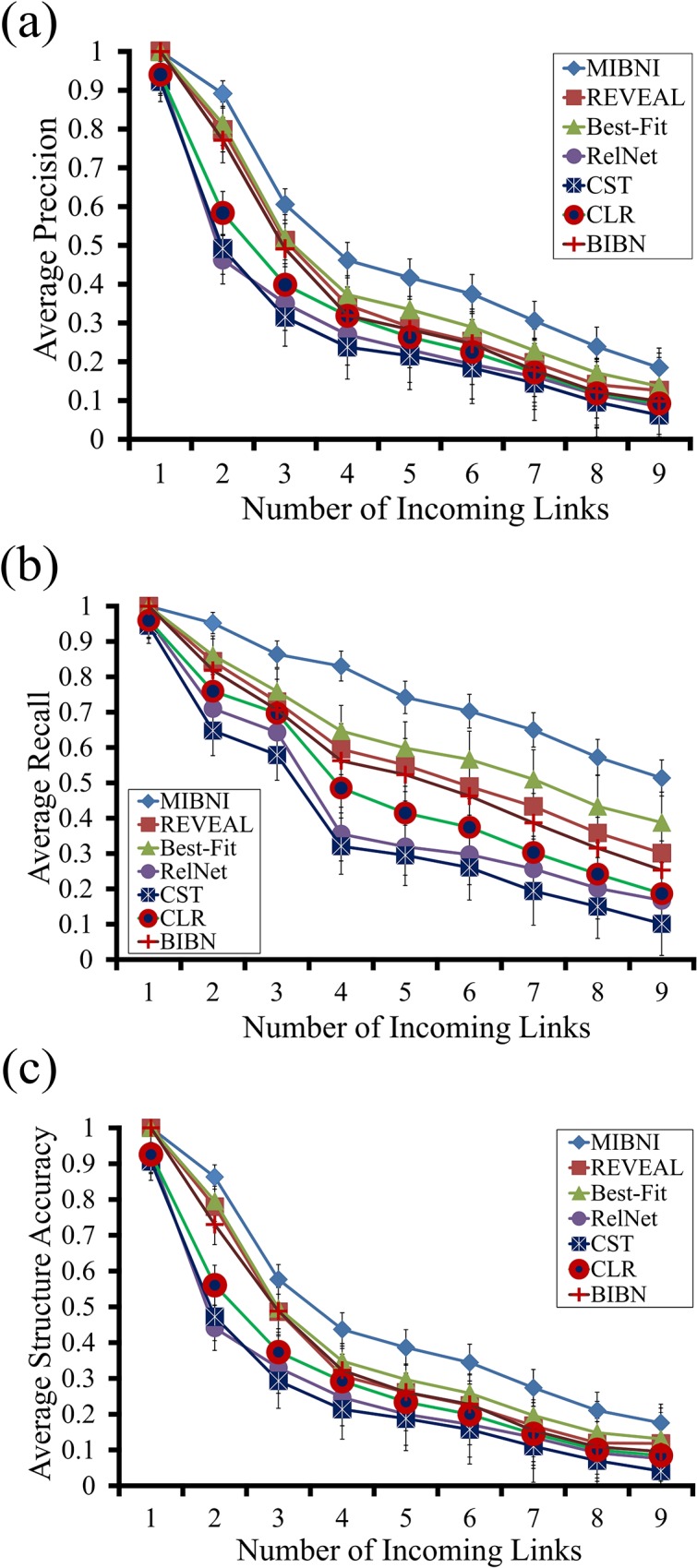
Comparison of precision, recall and structural accuracy between MIBNI and other methods in BA random networks. Results of **(a)** precision, **(b)** recall, and **(c)** structural accuracy, respectively. In each figure, 300 BA random networks with different network sizes (|V| = 10,20,…,100) were used as target networks, and a total of 16,500 nodes in those networks were classified into nine groups by the number of incoming links. The maximum time step was set to |V| + 10 in generating artificial gene expression data. Y-axis values show the average precision, recall, and structural accuracy values with respect to the target genes in each group. Error-bars mean the standard deviations. MIBNI showed the best performance in terms of precision, recall, and structural accuracy.

To compare performance, we applied MIBNI, REVEAL, Best-Fit, RelNet, CST, CLR, and BIBN to the artificial gene expression datasets generated from 300 BA random target networks and examined the structural accuracies with respect to the inferred networks (Fig 4). For more information, we classified all of the target genes into nine classes according to the number of incoming links (D), which ranged from 1 to 9. As shown in the figure, the average precision, recall, and structural accuracy values of all methods decreased as the number of incoming links increased. This is because the number of incoming links represents the degree of difficulty of the inference problem. Every method showed considerably higher structural performance in the easiest case of D = 1; in particular, MIBNI, REVEAL, Best-Fit and BIBN always found the correct regulatory genes in that case. However, MIBNI showed higher precision, recall, and structural accuracy values than all of the other methods in cases of D > 1. These results were consistently observed irrespective of the specific network size in BA random networks (see S2, S3, and S4 Figs) as well as GNW random networks (see S5, S6, and S7 Figs), which implies that the performance of MIBNI was highly stable.

#### Dynamics accuracy analysis

**Fig 5:**
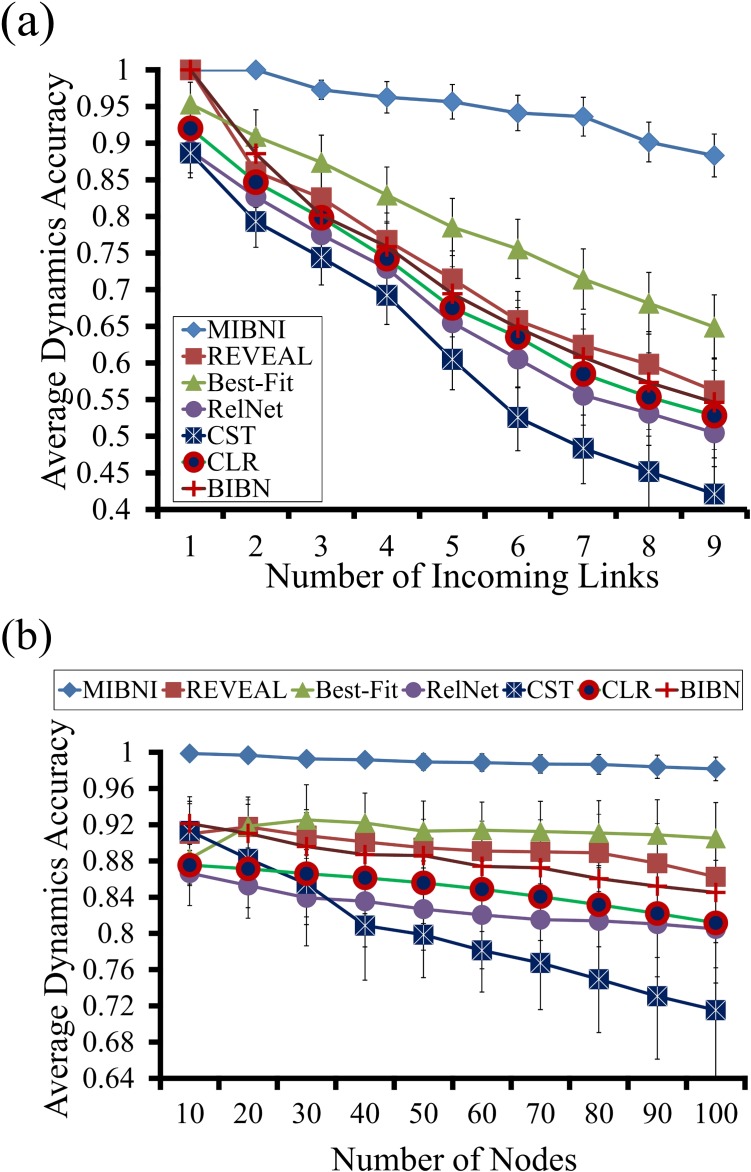
Comparison of dynamic accuracies between MIBNI and other methods in BA random networks. **(a)** Results versus the numbers of incoming links. A total of 300 BA random networks with different network sizes (|V| = 10,20,…,100) were used as target networks, and 16,500 nodes in those networks were classified according to the number of incoming links. **(b)** Results versus the network sizes. For each different number of nodes, 30 BA random networks were examined. Error-bars mean the standard deviations. In each figure, the maximum time step was set to |V| + 10 in generating artificial gene expression data.

We note that networks with different structures can produce the same dynamics. In this regard, it is also important to verify the network inference performance in terms of the dynamics accuracy. Therefore, we examined the dynamics accuracy (see Methods section for the definition) of the inferred networks by the MIBNI, REVEAL, Best-Fit, RelNet, CST, CLR and BIBN methods over the 300 BA random target networks (Fig 5). We note that the original REVEAL, Best-Fit, RelNet, CST, CLR and BIBN methods can determine only whether the regulatory interactions between genes exist or not, so we modified them in a way that they can additionally find the regulatory rules. To this end, we applied the “*search_update_rule*” subroutine shown in Fig 2B to the inferred regulatory interactions. As shown in Fig 5, we computed the average dynamics accuracy of the node groups as classified by the number of incoming links (Fig 5A; i.e., D = 1,2,3,…,9) and the network size (Fig 5B; i.e., |V| = 10,20,…,100). As seen in Fig 5A, the dynamics accuracies of all methods decreased as the number of incoming links increased. However, MIBNI showed significantly higher dynamics accuracy than all other methods, especially in cases with D > 1. This difference in accuracy increased as the number of incoming links increased. In particular, the dynamics accuracy of MIBNI was 1.0 (a perfect prediction) when D = 1 or 2. As shown in Fig 5B, MIBNI also exhibited the best dynamics accuracies irrespective of the network size. Specifically, the dynamics accuracies of MIBNI were higher than 0.9986 for all network sizes, whereas the maximal dynamics accuracies of REVEAL, Best-Fit, RelNet, CST, CLR, and BIBN were less than 0.9176, 0.9254, 0.8666, 0.9131, 0.8755, and 0.9213 respectively. This outstanding performance of MIBNI was consistently observed in simulations with GNW random networks (see S8 Fig). In addition, we examined the effect of a sample size of gene expression data on performance. To this end, we investigated changes of structural and dynamics accuracies by varying the maximum time step from 10 to 110 by 10 (see S9 Fig), and observed that both accuracies increased as the time step increased. In particular, the structural accuracy was more sensitive to the sample size than the dynamics accuracy.

#### Improvement by swapping

**Fig 6:**
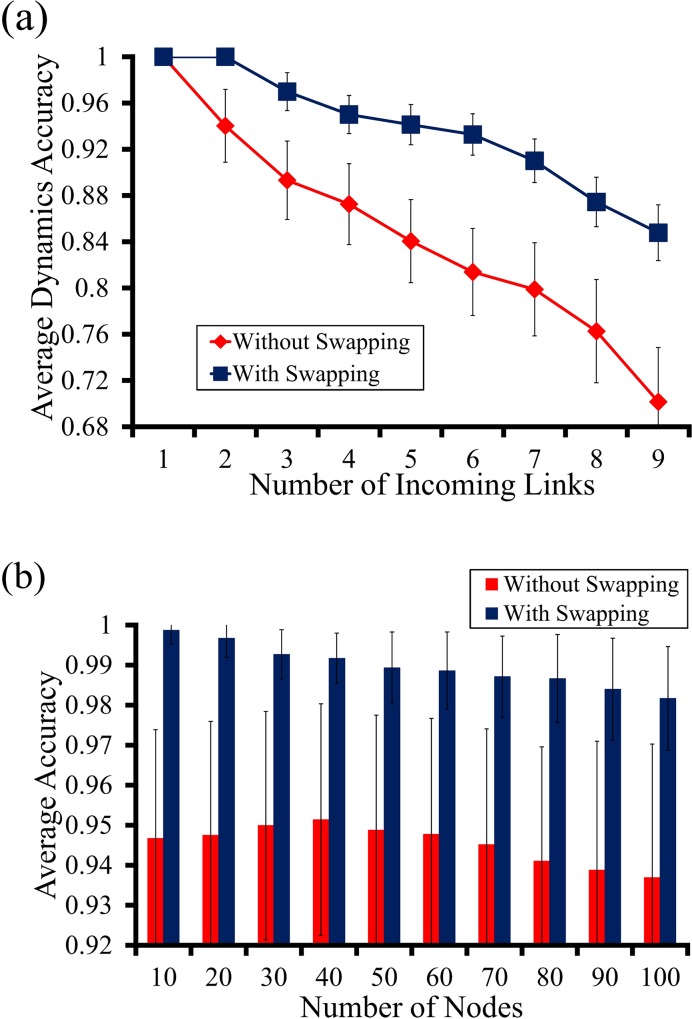
Performance improvements by SWAP subroutine. Two versions of MIBNI, in which the SWAP routine was included and not included, respectively, were compared with respect to dynamics accuracy. **(a)** Results versus the numbers of incoming links. Three hundred BA random networks with different network sizes (|V| = 10,20,…,100 and |A| = 2 ∙ |V|) used as target networks, and 16,500 nodes in those networks were classified according to the number of incoming links. **(b)** Results versus the network sizes. For each different number of nodes, 30 BA random networks were examined. The Y-axis value indicates the average dynamic accuracy of the nodes in each group, and error-bars mean the standard deviations. In each figure, the maximum time step was set to |V| + 10 in generating artificial gene expression data.

As previously mentioned, the MIFS approximates the multivariate mutual information value. The approximation error increases as the number of regulatory genes considered increases. To overcome this limitation, we inserted the SWAP subroutine into our MIBNI method. To show the performance improvement by SWAP, we compared the dynamics accuracies of MIBNI approaches with and without the SWAP subroutine over the 300 BA random networks (Fig 6). Herein, we call them “MIBNI_w/_SWAP” and “MIBNI_w/o_SWAP”, respectively, for convenience. In Fig 6A, MIBNI_w/_SWAP shows higher dynamic accuracy than MIBNI_w/o_SWAP for all D > 1 cases. The performance improvement by SWAP increased as the number of incoming links increased, indicating that SWAP is more efficient for relatively difficult problems. This result was consistently observed irrespective of the network size (see S10 Fig). We additionally examined the average performance improvement in network groups of different sizes (Fig 6B). The dynamics accuracies of MIBNI_w/_SWAP were consistently higher than those of MIBNI_w/o_SWAP were, irrespective of the network size. Thus, the SWAP subroutine plays an important role in improving the dynamic accuracy by ameliorating the approximation error caused by the MIFS subroutine.

#### Computation time comparison

**Fig 7:**
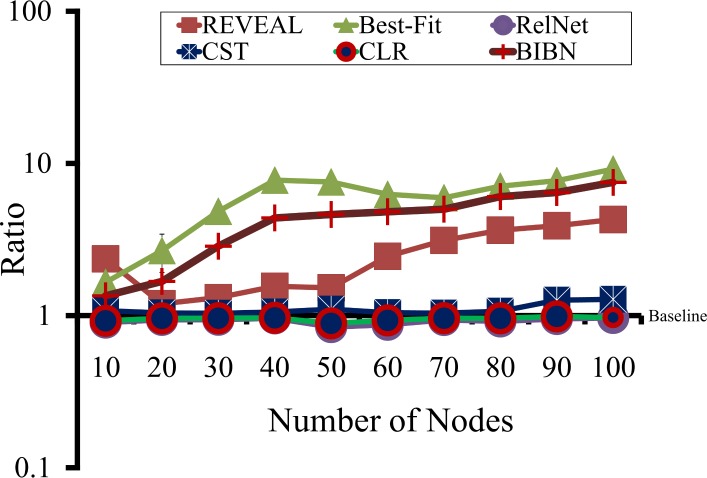
Comparison of the running times among the network inference methods. The Y-axis values represent the ratios of the average running times of six other methods over that of MIBNI. The Y = 1 line denotes the baseline for comparisons. The running times of Best-Fit, REVEAL and BIBN were significantly larger than that of MIBNI, whereas those of RelNet, CST, and CLR were similar to it.

The running times of most of the existing network inference algorithms are quite high and depend upon the incoming links of a target node in the network. We compared the running time of MIBNI with those of the REVEAL, Best-Fit, RelNet, CST, CLR and BIBN methods over the 300 BA random networks in Fig 7. In the figure, the Y-axis values are the ratios of the average running times of the compared methods over that of MIBNI. Thus, Y = 1 is the baseline for comparisons. The running time of MIBNI was significantly smaller than those of REVEAL, Best-Fit and BIBN were, and was similar to those of RelNet, CST, and CLR. We must note that the latter three methods showed the worst performance in terms of structural and dynamics accuracy, as seen in Figs 4 and 5. In other words, the improved speeds of RelNet, CST, and CLR were obtained by sacrificing their prediction accuracies.

### Performance on real networks

To validate the performance of MIBNI, we tested it with two real gene expression datasets from an *E*. *coli* gene regulatory network and a fission yeast cell cycle network (see S11 Fig).

#### Case study 1: E. coli network

**Fig 8:**
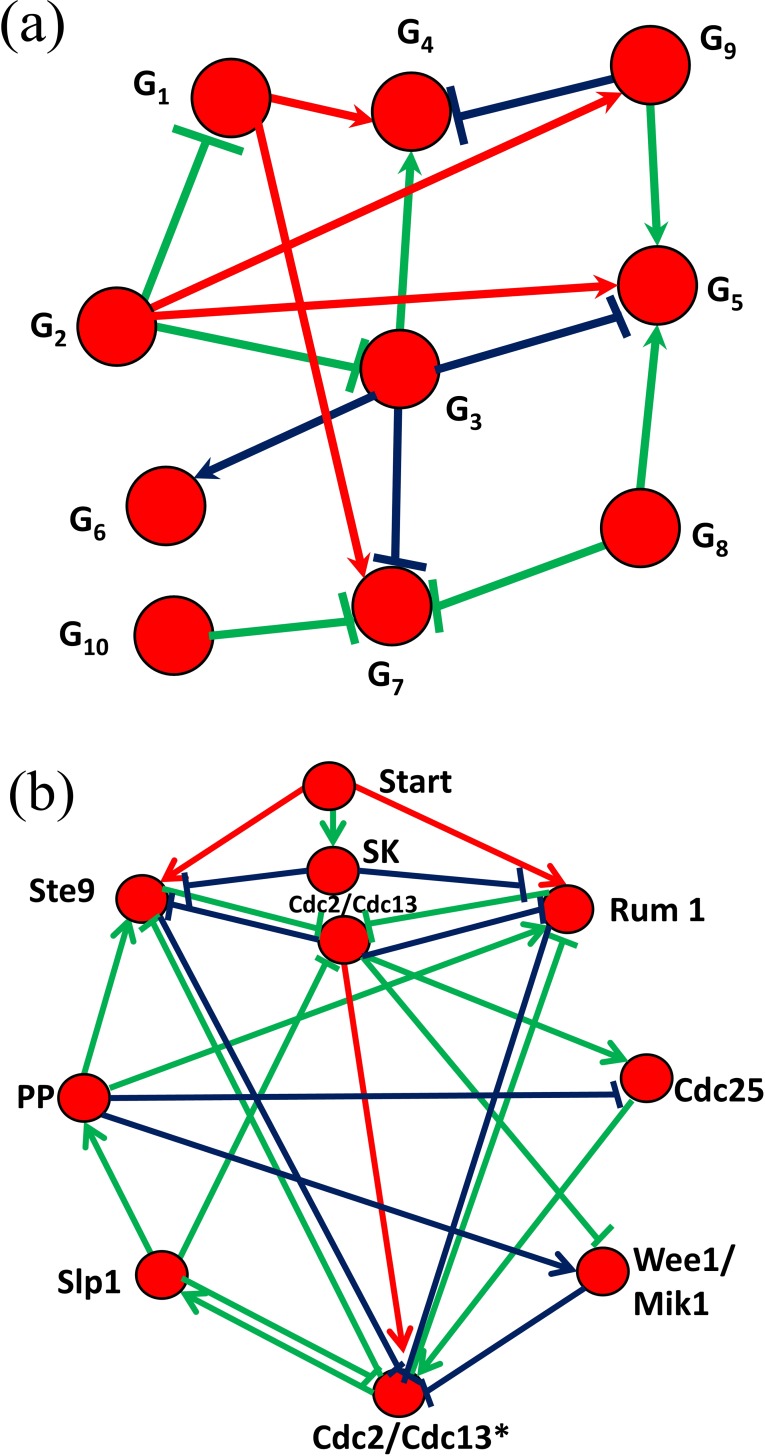
Inference performance of MIBNI with two real biological network datasets. The green, red, and blue interactions denote true positive, false positive, and false negative predictions, respectively. **(a)** Inference result of an *E*. *coli* gene regulatory network consisting of 10 genes and 11 interactions. The maximum time step of the gene expression data was 21. The predicted result shows 7 true positives, 4 false positives, and 4 false negatives. The structural and dynamics accuracies were 0.9200 and 0.9700, respectively. **(b)** Inference result of a fission yeast cell cycle network consisting of 10 genes and 23 interactions. The maximum time step of the gene expression data was 10. The predicted result shows 14 true positives, 3 false positives, and 9 false negatives. The structural and dynamics accuracies were 0.8800 and 0.9800, respectively.

Our method was tested with a time-series gene expression dataset used in the DREAM3 challenge [24], which was derived from an *E*. *coli* gene regulatory network consisting of 10 nodes and 11 interactions. The original gene expression data was represented by real values (see S2 Table), so it was converted to another dataset of Boolean values using a *K*-means clustering algorithm-based discretization method [17] (see S3 Table). Then, MIBNI inferred a network, as shown in Fig 8A, by correctly constructing 7 out of 11 interactions. On the other hand, REVEAL, Best-Fit, CST, RelNet, CLR and BIBN correctly inferred 4, 5, 2, 3, 4 and 4 interactions, respectively (see S12 Fig). We also found that the dynamics accuracy of MIBNI was 0.9700, whereas those of REVEAL, Best-Fit, CST, RelNet, CLR and BIBN were 0.9300, 0.9500, 0.8900, 0.8700, 0.8100, and 0.9200 respectively. In other words, MIBNI showed the best performance in terms of both structural and dynamics accuracies. We also note that the dynamics accuracy was relatively high considering the structural accuracy. This is because networks with different structures can induce the same dynamics. For example, gene G_2_ was inferred as a regulatory gene for gene G_5_ by MIBNI, whereas G_3_ is the real regulatory gene in the real network (Fig 8A). Despite this incorrectly inferred structure, the time-series expression of gene G_5_ was predicted with no error. It is also interesting that MIBNI uniquely inferred two interactions G_3_–G_4_ and G_9_–G_5_ which are not inferred by any of six other methods. On the other hand, there was no interaction uniquely inferred by either of REVEAL, Best-Fit, RelNet, CLR, or BIBN, and only one interaction G_3_–G_7_ uniquely inferred by CST. We also need to note a previous result [25] where a time-delayed dynamic Bayesian network inference method was used to infer the same *E*. *coli* network used in this study. Although it inferred the same number of true positive interactions as MIBNI, it utilized additional information such as heterozygous knockdown and null mutant knockout data.

#### Case study 2: Yeast cell cycle network

As another case study, we considered a fission yeast cell cycle network consisting of 10 nodes and 23 interactions that was used in a previous study [26], which provided a time-series gene expression dataset of Boolean values (see S4 Table). As shown in Fig 8B, MIBNI correctly inferred 14 out of 23 interactions. In contrast, REVEAL, Best-Fit, RelNet, CST, CLR and BIBN correctly inferred 10, 12, 4, 11, 12, and 10 interactions, respectively (see S13 Fig). We also found that the dynamics accuracy of MIBNI was 0.9800, whereas those of REVEAL, Best-Fit, RelNet, CST, CLR, and BIBN were 0.9200, 0.8800, 0.8700, 0.8600, 0.8400, and 0.9000 respectively. As in the *E*. *coli* case study, MIBNI showed significantly better performance than did any of the other methods in terms of structural and dynamics accuracies. In addition, MIBNI uniquely inferred two interactions, cdc2/cdc13 – ste9 and cdc2/cdc13 – rum1, whereas none of six other methods uniquely inferred any interaction. This can be a useful characteristics in network inference.

## Conclusions

The inference of a hidden gene regulatory network from time-series gene expression data is a challenging problem in systems biology. Nevertheless, there have been few methods proposed for that problem, as researchers have focused more on the accurate prediction of the network dynamics than that of the network structure. In this regard, we proposed a novel mutual information-based Boolean network inference method called MIBNI, in which a Boolean network model with a restricted update scheme to capture coarse-grained dynamics efficiently was employed. MIBNI repeatedly executes two main subroutines, MIFS and SWAP. The MIFS subroutine identifies an initial set of regulatory genes for each target gene using mutual information-based feature selection. Then, the SWAP subroutine improves the dynamics prediction accuracy by iteratively swapping a pair of genes between the set of the selected regulatory genes and the set of other genes. Through extensive simulations with artificial gene expression datasets, MIBNI showed consistently better performance than REVEAL, Best-Fit, RelNet, CST, CLR, or BIBN in terms of both structural and dynamics prediction accuracies. Moreover, MIBNI was significantly faster than REVEAL and Best-Fit. In addition, our method achieved good performance with two real gene expression datasets for an *E*. *coli* gene regulatory network and a fission yeast cell cycle network. Based on these results, we suggest that MIBNI is a promising tool for predicting both the network structure and the dynamics of a gene regulatory network. One limitation of MIBNI is the update scheme, which assumes that the update rule is represented only by a conjunctive or disjunctive function. Our future research will improve MIBNI by using a more generalized update scheme. Another limitation is about the parameter of the maximum number of incoming links to be inferred. Although a considerably larger value was specified in MIBNI than in previous inference methods, there is no general criteria to efficiently determine the parameter value. The last limitation is that our method is a local optimization approach. Considering a tradeoff between computational time and inference accuracy, there is room to improve the inference accuracy performance by sacrificing the computation time. For example, MIBNI can be modified into a global optimization method by replacing the MIFS subroutine with one of various meta-heuristics such as simulated annealing, genetic algorithms, swarm-based optimization, and tabu-search, which are time-consuming but eventually help to avoid trapping into local optimum solutions. This modification can be also one of the interesting future studies.

## Supporting information

S1 Fig Pseudo-code of the Barabási-Albert model used in our simulation. (PDF)

S2 Fig Comparison of precision between MIBNI and other methods in BA random network groups of different sizes. (PDF)

S3 Fig Comparison of recall between MIBNI and other methods in BA random network groups of different sizes. (PDF)

S4 Fig Comparison of structural accuracy between MIBNI and other methods in BA random network groups of different sizes. (PDF)

S5 Fig Comparison of precision between MIBNI and other methods in GNW random network groups of different sizes. (PDF)

S6 Fig Comparison of recall between MIBNI and other methods in GNW random network groups of different sizes. (PDF)

S7 Fig Comparison of structural accuracy between MIBNI and other methods in GNW random network groups of different sizes. (PDF)

S8 Fig Comparison of dynamic accuracies between MIBNI and other methods in GNW random networks. (PDF)

S9 Fig Changes of structural and dynamics accuracy of MIBNI in BA random network groups against the number of samples. (PDF)

S10 Fig Performance improvement by SWAP subroutine in BA random network groups of different sizes. (PDF)

S11 Fig Structures of two real regulatory networks. (PDF)

S12 Fig Inference performance of REVEAL, Best-Fit, CST, RelNet, CLR, and BIBN with respect to the *E. coli* gene regulatory network. (PDF)

S13 Fig Inference performance of REVEAL, Best-Fit, CST, RelNet, CLR, and BIBN with respect to fission yeast cell cycle network. (PDF)

S1 Table The number of nodes and the range of the number of edges in GNW random networks. (PDF)

S2 Table Real-valued gene expression dataset of the *E.coli* gene regulatory network. (PDF)

S3 Table Boolean gene expression dataset of the *E. coli* gene regulatory network converted by *K*-means algorithm. (PDF)

S4 Table Boolean gene expression dataset of the fission yeast cell cycle. (PDF)

## Abbreviations

BN: Boolean network
MIFS: mutual information-based feature selection
MIBNI: mutual information-based network inference
REVEAL: reverse engineering algorithm
CLR: context likelihood of relatedness
CST: chi-square test
RelNet: relevance network
BIBN: Bayesian inference approach for a Boolean network
