# Supplementary material for: A novel mutual information-based Boolean network inference method from time-series gene expression data
Source: PLoS One. 2017 Feb 8;12(2):e0171097. doi: 10.1371/journal.pone.0171097 (PMC5298315; doi:10.1371/journal.pone.0171097)
Supplement: S1 Fig — (PDF) [file pone.0171097.s001.pdf]

```
function [V, A] = createRBN_BarabasiAlbert(N, e, d)
    //N:   The desired number of nodes
    //e:   The number of initial nodes
    //d:   The number of interactions to be added at each step
    //V, A: A set of nodes V and a set of links A of the resulting network generated by the Barabási-Albert model

    V←{0, 1, …, e-1};
    A←∅;
    for i:=0 to e-2
        for j:=i+1 to e-1
            if (randNumber(0,1) < 0.5)  // randNumber(0,1) returns a real number chosen from 0 to 1 uniformly at random
                A←A∪{(i, j)};
            else
                A←A∪{(j, i)};
            endif
        endfor
    endfor

    for i:= e to N-1
        for j:=0 to d-1
            do
                v = selection(V); //v is chosen with a probability proportional to its degree.
                if (randNumber(0,1) < 0.5)
                    vSrc← i, vDst← v;
                else
                    vSrc← v, vDst← i;
                endif
            until ((vSrc, vDst)∉ A);
            A←A∪{(vSrc, vDst)};
        endfor
        V←V∪{i};
    endfor
    return [V, A];
end
```

S1 Figure. **Pseudo-code of the Barabási-Albert model used in our simulation**. The desired number of nodes (N), the number of nodes in the seed network (e), and the number of edges to be added at each iteration (d) are given as parameters. A small seed network $G(V, A)$ consisting of $V = \{v_1, v_2, \ldots\ldots, v_e\}$ and $A = \{(v_i, v_j) \mid i, j = 1,2, \ldots, e, and\ i \neq j\}$ is randomly specified. At each iteration, a node v and d different interactions are newly inserted into the graph where the probability of connecting a new node and an existing node is proportional to the connectivity of the latter node. This process is repeated until $|V| = N$ and a resultant network is returned as the output.
