# Supplementary material for: A novel mutual information-based Boolean network inference method from time-series gene expression data
Source: PLoS One. 2017 Feb 8;12(2):e0171097. doi: 10.1371/journal.pone.0171097 (PMC5298315; doi:10.1371/journal.pone.0171097)
Supplement: S5 Fig — (PDF) [file pone.0171097.s005.pdf]

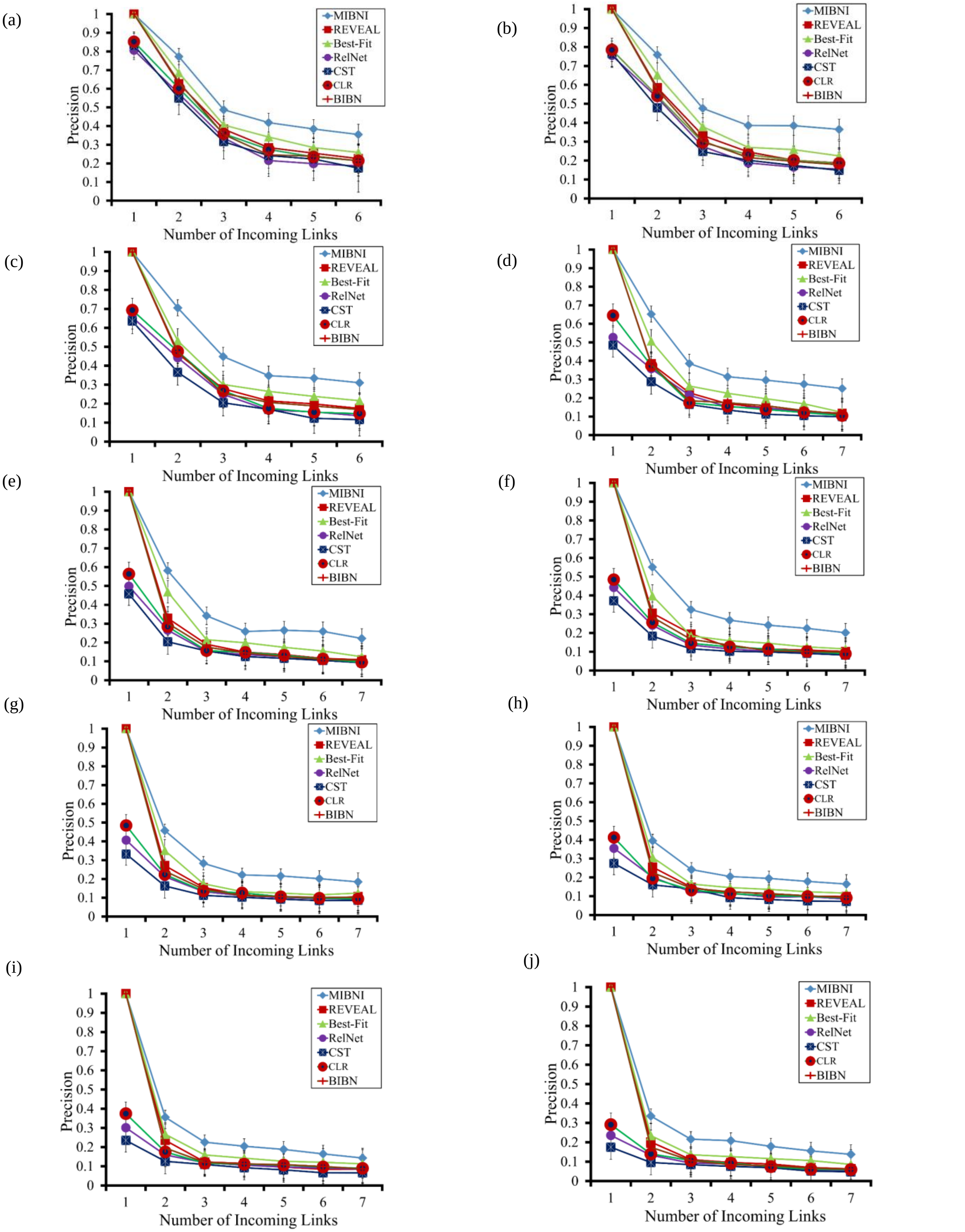

S5 Figure. Comparison of precision between MIBNI and other methods in GNW random network groups of different sizes. (a)-(j) Precision results of network groups with $|V| = 10, 20, \ldots, 100$, respectively. In each subfigure, 30 GNW random networks were examined.
