# Supplementary material for: A novel mutual information-based Boolean network inference method from time-series gene expression data
Source: PLoS One. 2017 Feb 8;12(2):e0171097. doi: 10.1371/journal.pone.0171097 (PMC5298315; doi:10.1371/journal.pone.0171097)
Supplement: S8 Fig — (PDF) [file pone.0171097.s008.pdf]

(a)

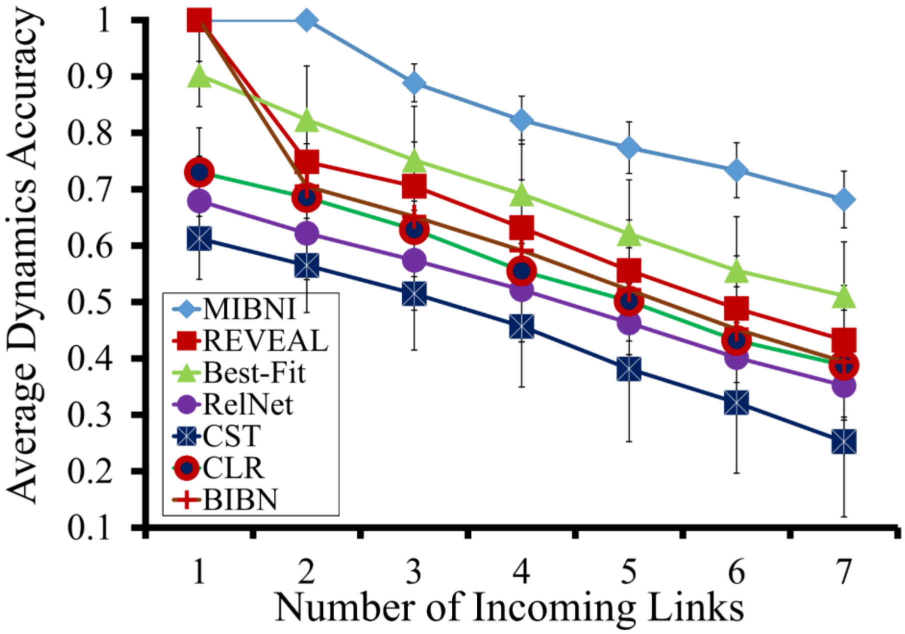

(b)

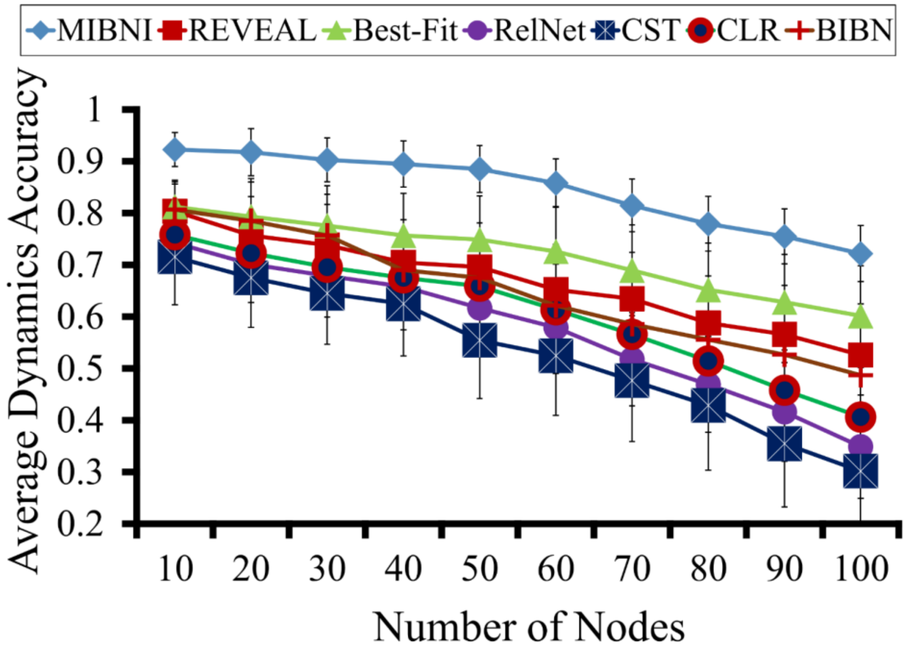

S8 Figure. Comparison of dynamic accuracies between MIBNI and other methods in GNW random networks. **(a)** Results versus the numbers of incoming links. A total of 300 GNW random networks with different network sizes ($|V| = 10, 20, \ldots, 100$) were used as target networks, and 16,500 nodes in those networks were classified according to the number of incoming links. **(b)** Results versus the network sizes. For each different number of nodes, 30 GNW random networks were examined. In each figure, the maximum time step was set to $|V| + 10$ in generating artificial gene expression data.
