# Supplementary material for: A novel mutual information-based Boolean network inference method from time-series gene expression data
Source: PLoS One. 2017 Feb 8;12(2):e0171097. doi: 10.1371/journal.pone.0171097 (PMC5298315; doi:10.1371/journal.pone.0171097)
Supplement: S9 Fig — (PDF) [file pone.0171097.s009.pdf]

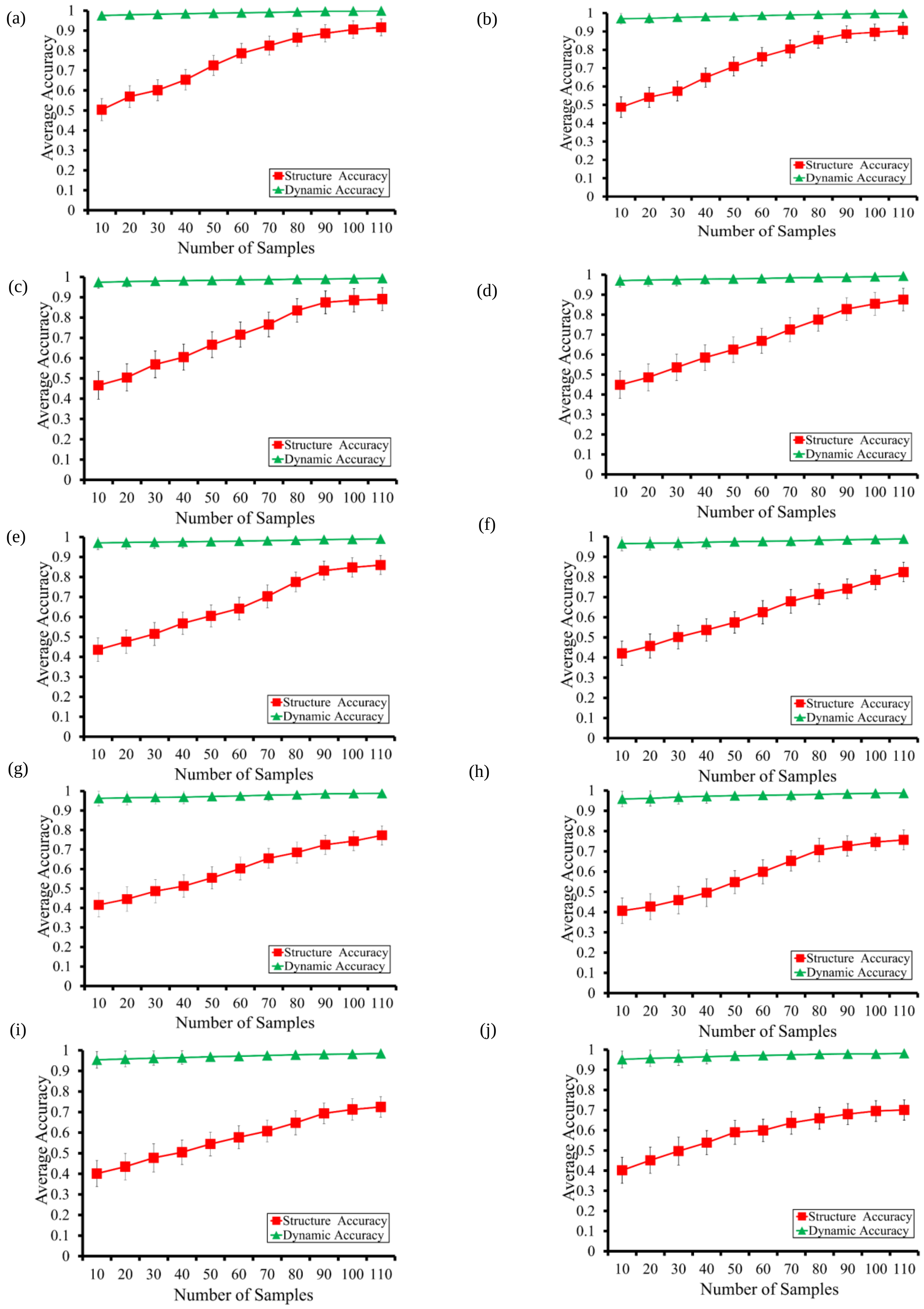

S9 Figure. Changes of structural and dynamics accuracy of MIBNI in BA random network groups against the number of samples. (a)-(j) Structural accuracy results of network groups with $|V| = 10, 20, \dots, 100$, respectively. The number of links ($|A|$) was set to $2 \cdot |V|$ in this work, and 30 BA random networks were examined in each subfigure. The maximum time step (i.e., the number of samples) in gene expression datasets varies from 10 to 110 by 10.
