# Supplementary material for: A novel mutual information-based Boolean network inference method from time-series gene expression data
Source: PLoS One. 2017 Feb 8;12(2):e0171097. doi: 10.1371/journal.pone.0171097 (PMC5298315; doi:10.1371/journal.pone.0171097)
Supplement: S10 Fig — (PDF) [file pone.0171097.s010.pdf]

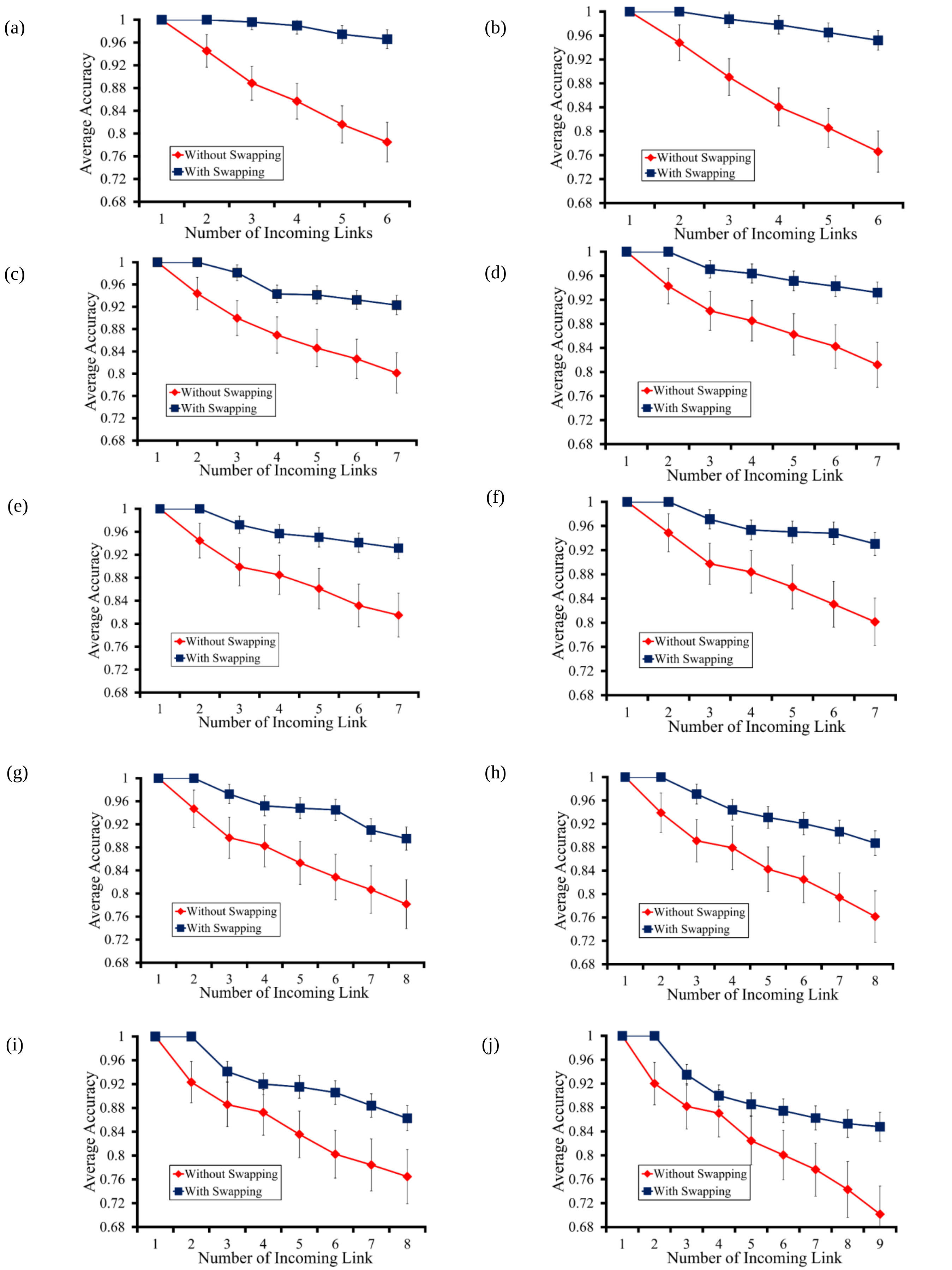

S10 Figure. Performance improvement by SWAP subroutine in BA random network groups of different sizes. (a)-(j) Results of network groups with $|V| = 10, 20, \ldots, 100$, respectively. The number of links ($|A|$) was set to $2 \cdot |V|$, and 30 BA random networks were examined in each subfigure. The Y-axis values show changes in the average dynamic accuracies versus the number of incoming links.
