# Supplementary material for: A novel mutual information-based Boolean network inference method from time-series gene expression data
Source: PLoS One. 2017 Feb 8;12(2):e0171097. doi: 10.1371/journal.pone.0171097 (PMC5298315; doi:10.1371/journal.pone.0171097)
Supplement: S11 Fig — (PDF) [file pone.0171097.s011.pdf]

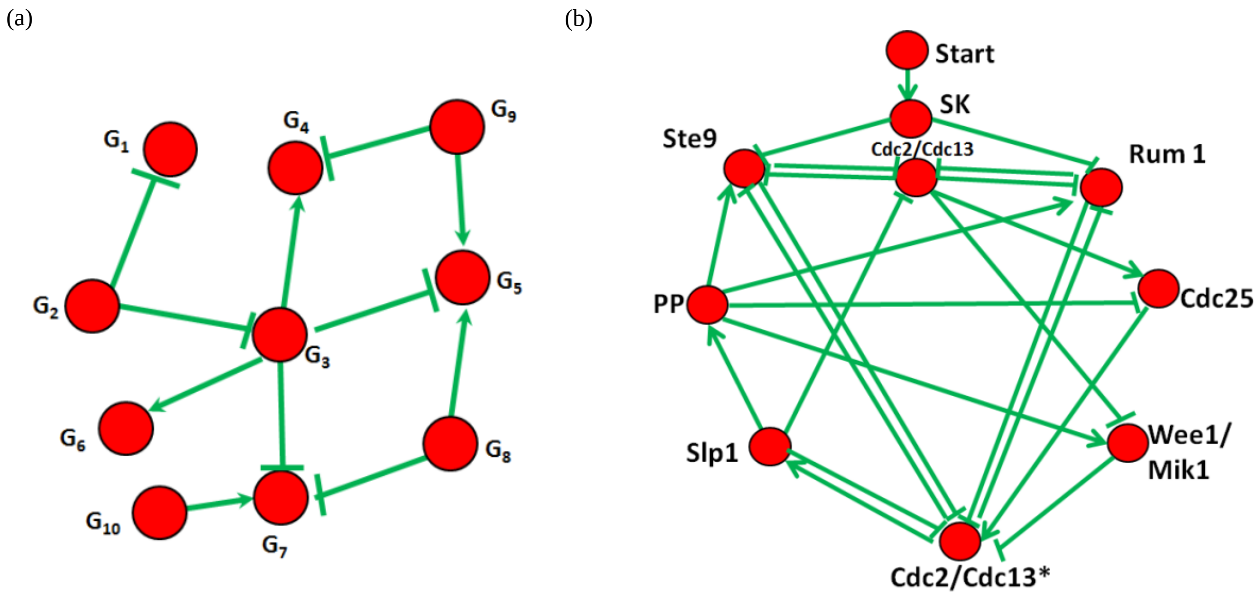

S11 Figure. Structures of two real regulatory networks. (a) The *E. coli* gene regulatory network consisting of 10 genes and 11 interactions. **(b)** The fission yeast cell cycle network consisting of 10 genes and 23 interactions. In the figure, the arrows and barred lines represent positive and negative interactions, respectively.
