# Supplementary material for: A novel mutual information-based Boolean network inference method from time-series gene expression data
Source: PLoS One. 2017 Feb 8;12(2):e0171097. doi: 10.1371/journal.pone.0171097 (PMC5298315; doi:10.1371/journal.pone.0171097)
Supplement: S12 Fig — (PDF) [file pone.0171097.s012.pdf]

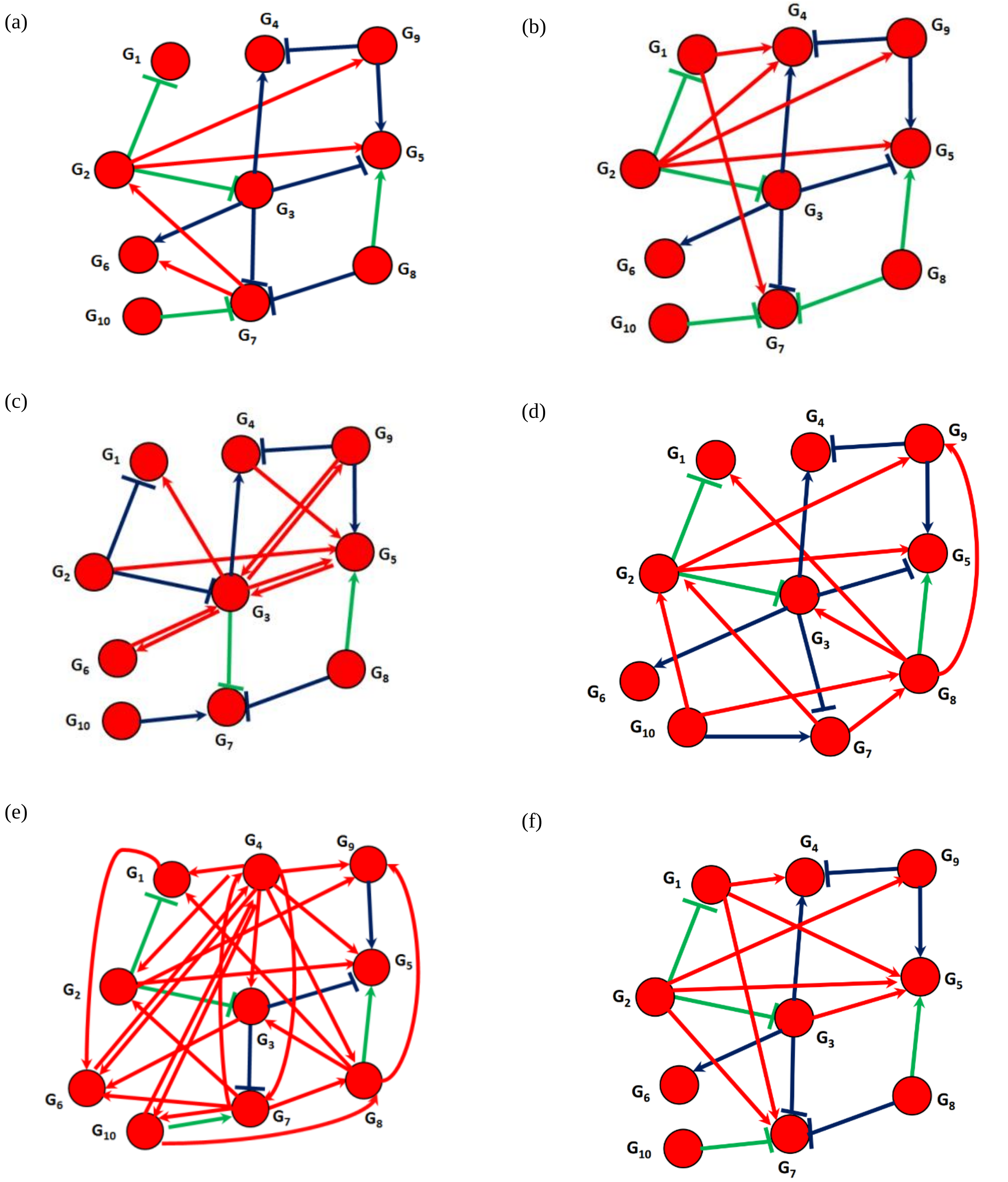

S12 Figure. Inference performance of REVEAL, Best-Fit, CST, RelNet, CLR, and BIBN with respect to the E. coli gene regulatory network. The green, red, and blue interactions denote true positive, false positive, and false negative predictions, respectively. **(a)** Inference results of REVEAL. The results showed 4 true positives, 4 false positives, and 7 false negatives. The structural and dynamics accuracies were 0.8865 and 0.9300, respectively. **(b)** Inference results of Best-Fit. The results showed 5 true positives, 5 false positives, and 6 false negatives. The structural and dynamics accuracies were 0.8865 and 0.9500, respectively. **(c)** Inference results of CST. The results showed 2 true positives, 9 false positives, and 7 false negatives. The structural and dynamics accuracies were 0.8315 and 0.8900, respectively. **(d)** Inference results of RelNet. The results showed 3 true positives, 9 false positives, and 5 false negatives. The structural and dynamics accuracies were 0.8526 and 0.8700, respectively. **(e)** Inference results of CLR. The results showed 4 true positives, 24 false positives, and 3 false negatives. The structural and dynamics accuracies were 0.7096 and 0.8100, respectively. **(f)** Inference results of BIBN. The results showed 4 true positives, 7 false positives, and 6 false negatives. The structural and dynamics accuracies were 0.8645 and 0.9200, respectively.
