# Supplementary material for: A novel mutual information-based Boolean network inference method from time-series gene expression data
Source: PLoS One. 2017 Feb 8;12(2):e0171097. doi: 10.1371/journal.pone.0171097 (PMC5298315; doi:10.1371/journal.pone.0171097)
Supplement: S13 Fig — (PDF) [file pone.0171097.s013.pdf]

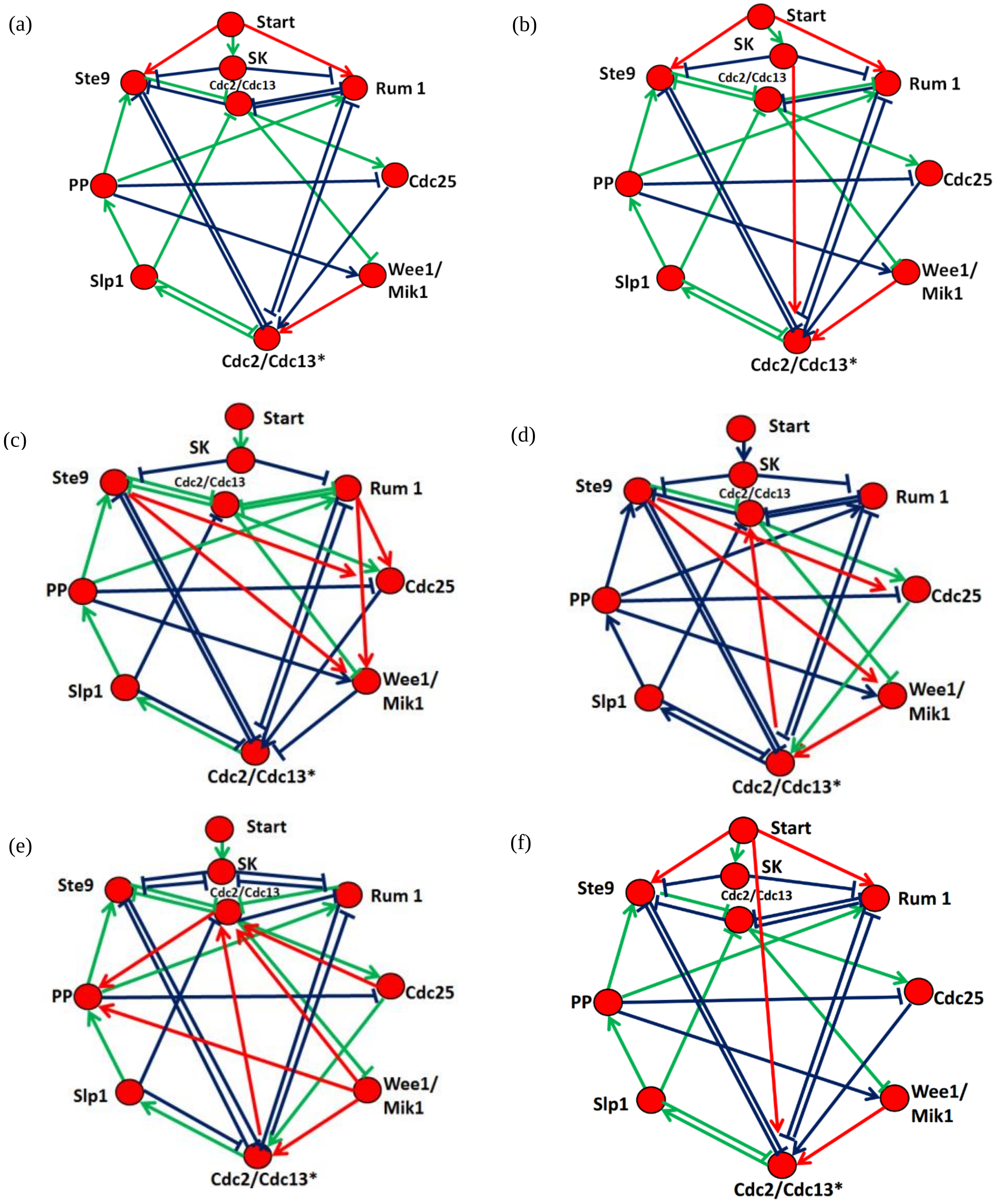

S13 Figure. Inference performance of REVEAL, Best-Fit, CST, RelNet, CLR, and BIBN with respect to fission yeast cell cycle network. The green, red, and blue interactions denote true positive, false positive, and false negative predictions, respectively. **(a)** Inference results of REVEAL. The results showed 10 true positives, 3 false positives, and 12 false negatives. The structural and dynamics accuracies were 0.8529 and 0.9200, respectively. **(b)** Inference results of Best-Fit. The results showed 12 true positives, 4 false positives, and 10 false negatives. The structural and dynamics accuracies were 0.8585 and 0.8800, respectively. **(c)** Inference results of CST. The results showed 11 true positives, 4 false positives, and 12 false negatives. The structural and dynamics accuracies were 0.8431 and 0.8600, respectively. **(d)** Inference results of RelNet. The results showed 4 true positives, 4 false positives, and 18 false negatives. The structural and dynamics accuracies were 0.7962 and 0.8700, respectively. **(e)** Inference results of CLR. The results showed 11 true positives, 12 false positives, and 12 false negatives. The structural and dynamics accuracies were 0.7647 and 0.8400, respectively. **(f)** Inference results of BIBN. The results showed 10 true positives, 4 false positives, and 12 false negatives. The structural and dynamics accuracies were 0.8461 and 0.9000, respectively.
