# Supplementary material for: A novel mutual information-based Boolean network inference method from time-series gene expression data
Source: PLoS One. 2017 Feb 8;12(2):e0171097. doi: 10.1371/journal.pone.0171097 (PMC5298315; doi:10.1371/journal.pone.0171097)
Supplement: S1 Table — (PDF) [file pone.0171097.s014.pdf]

**S1 Table.** The number of nodes and the range of the number of edges in GNW random networks.

| Number of nodes | Range of the number of edges |
|---|---|
| 10 | 27-29 |
| 20 | 40-58 |
| 30 | 55-76 |
| 40 | 75-86 |
| 50 | 93-114 |
| 60 | 100-137 |
| 70 | 134-150 |
| 80 | 151-169 |
| 90 | 177-194 |
| 100 | 188-204 |
