# Supplementary material for: A novel mutual information-based Boolean network inference method from time-series gene expression data
Source: PLoS One. 2017 Feb 8;12(2):e0171097. doi: 10.1371/journal.pone.0171097 (PMC5298315; doi:10.1371/journal.pone.0171097)
Supplement: S2 Table — (PDF) [file pone.0171097.s015.pdf]

S2 Table. Real-valued gene expression dataset of the *E.coli* gene regulatory network

Time	G_1	G_2	G_3	G_4	G_5	G_6	G_7	G_8	G_9	G_{10}
1	0.502070	0.657391	0.663857	0.686757	0.418227	0.194776	0.940575	0.695161	0.657907	0.823619
2	0.428431	0.721075	0.55796	0.67999	0.485694	0.258959	0.775269	0.758334	0.733297	0.897461
3	0.366991	0.772921	0.469909	0.673257	0.54135	0.310081	0.646417	0.808416	0.792073	0.940389
4	0.315855	0.81513	0.396972	0.666344	0.58717	0.350777	0.547216	0.848119	0.837896	0.965346
5	0.273351	0.849494	0.336699	0.659061	0.6249	0.383129	0.472071	0.879594	0.87362	0.979854
6	0.238049	0.87747	0.286972	0.651251	0.656044	0.408757	0.416374	0.904547	0.901472	0.988288
7	0.208745	0.900246	0.245993	0.642793	0.681886	0.428895	0.376309	0.924328	0.923185	0.993191
8	0.184426	0.918788	0.212253	0.633614	0.703521	0.444419	0.348693	0.94001	0.940113	0.996042
9	0.16425	0.933884	0.184489	0.623693	0.721886	0.455867	0.330868	0.952442	0.953311	0.997699
10	0.147514	0.946173	0.161656	0.613063	0.737774	0.463441	0.320612	0.962298	0.9636	0.998662
11	0.133634	0.956179	0.142884	0.601811	0.751849	0.467027	0.31608	0.970111	0.971622	0.999222
12	0.122123	0.964324	0.127456	0.590068	0.764642	0.466273	0.315757	0.976305	0.977876	0.999548
13	0.112577	0.970955	0.114779	0.578001	0.776553	0.46075	0.318417	0.981215	0.982752	0.999737
14	0.104662	0.976354	0.104366	0.565795	0.787855	0.450192	0.323087	0.985108	0.986553	0.999847
15	0.098099	0.98075	0.095813	0.55364	0.798703	0.434728	0.329009	0.988194	0.989516	0.999911
16	0.092658	0.984328	0.088789	0.541716	0.809157	0.414983	0.335607	0.990641	0.991827	0.999948
17	0.088147	0.987241	0.083022	0.530184	0.819209	0.391999	0.342458	0.99258	0.993628	0.99997
18	0.084407	0.989613	0.078287	0.519176	0.828813	0.367028	0.349257	0.994118	0.995032	0.999983
19	0.081307	0.991543	0.074399	0.508795	0.837905	0.341303	0.355798	0.995337	0.996127	0.999998
20	0.078737	0.993115	0.071209	0.499112	0.846428	0.315877	0.361946	0.996303	0.996981	0.999994
21	0.076606	0.994395	0.06859	0.49017	0.854335	0.291544	0.367623	0.997069	0.997646	0.999997
