# Supplementary material for: A novel mutual information-based Boolean network inference method from time-series gene expression data
Source: PLoS One. 2017 Feb 8;12(2):e0171097. doi: 10.1371/journal.pone.0171097 (PMC5298315; doi:10.1371/journal.pone.0171097)
Supplement: S3 Table — (PDF) [file pone.0171097.s016.pdf]

S3 Table. Boolean gene expression dataset of the *E. coli* gene regulatory network converted by k-means algorithm

Time	G_1	G_2	G_3	G_4	G_5	G_6	G_7	G_8	G_9	G_{10}
1	1	0	1	1	0	0	1	0	0	0
2	1	0	1	1	0	0	1	0	0	0
3	1	0	1	1	0	0	1	0	0	0
4	1	0	1	1	0	0	0	0	0	1
5	1	1	1	1	0	1	0	1	0	1
6	0	1	0	1	1	1	0	1	1	1
7	0	1	0	1	1	1	0	1	1	1
8	0	1	0	1	1	1	0	1	1	1
9	0	1	0	1	1	1	0	1	1	1
10	0	1	0	1	1	1	0	1	1	1
11	0	1	0	1	1	1	0	1	1	1
12	0	1	0	0	1	1	0	1	1	1
13	0	1	0	0	1	1	0	1	1	1
14	0	1	0	0	1	1	0	1	1	1
15	0	1	0	0	1	1	0	1	1	1
16	0	1	0	0	1	1	0	1	1	1
17	0	1	0	0	1	0	0	1	1	1
18	0	1	0	0	1	0	0	1	1	1
19	0	1	0	0	1	0	0	1	1	1
20	0	1	0	0	1	0	0	1	1	1
21	0	1	0	0	1	0	0	1	1	1
