# Supplementary material for: A novel mutual information-based Boolean network inference method from time-series gene expression data
Source: PLoS One. 2017 Feb 8;12(2):e0171097. doi: 10.1371/journal.pone.0171097 (PMC5298315; doi:10.1371/journal.pone.0171097)
Supplement: S4 Table — (PDF) [file pone.0171097.s017.pdf]

S4 Table. Boolean gene expression dataset of the fission yeast cell cycle

Time	Start	SK	Cdc2/Cdc13	Ste9	Rum1	Slp1	Cdc2/Cdc13*	Week1Mik1	Cdc25	PP	Phase
1	1	0	0	1	1	0	0	1	0	0	START
2	0	1	0	1	1	0	0	1	0	0	G1
3	0	0	0	0	0	0	0	1	0	0	G1/S
4	0	0	1	0	0	0	0	1	0	0	G2
5	0	0	1	0	0	0	0	0	1	0	G2
6	0	0	1	0	0	0	1	0	1	0	G2/M
7	0	0	1	0	0	1	1	0	1	0	G2/M
8	0	0	0	0	0	1	0	0	1	1	M
9	0	0	0	1	1	0	0	1	0	1	M
10	0	0	0	1	1	0	0	1	0	0	G1
